# Deep learning radiomics of elastography for diagnosing compensated advanced chronic liver disease: an international multicenter study

**DOI:** 10.1186/s42492-025-00199-6

**Published:** 2025-08-15

**Authors:** Xue Lu, Haoyan Zhang, Hidekatsu Kuroda, Matteo Garcovich, Victor de Ledinghen, Ivica Grgurević, Runze Linghu, Hong Ding, Jiandong Chang, Min Wu, Cheng Feng, Xinping Ren, Changzhu Liu, Tao Song, Fankun Meng, Yao Zhang, Ye Fang, Sumei Ma, Jinfen Wang, Xiaolong Qi, Jie Tian, Xin Yang, Jie Ren, Ping Liang, Kun Wang

**Affiliations:** 1 Department of Ultrasound, Guangdong Key Laboratory of Liver Disease Research, Third Affiliated Hospital of Sun Yat-Sen University, Guangzhou, 510630 Guangdong China; 2 CAS Key Laboratory of Molecular Imaging, Institute of Automation, Chinese Academy of Sciences, Beijing, 100190 China; 3 School of Artificial Intelligence, University of Chinese Academy of Sciences, Beijing, 100190 China; 4 Division of Hepatology, Department of Internal Medicine, School of Medicine, Iwate Medical University, Shiwa-Gun, Iwate, 028-3694 Japan; 5 Medicina Interna E Gastroenterologia, Fondazione Policlinico Universitario Agostino Gemelli IRCCS, Università Cattolica del Sacro Cuore, Rome, 00168 Italy; 6 Hepatology Unit, University Hospital, CHU Bordeaux, Pessac, & INSERM U1312, Bordeaux University, Bordeaux, 33000 France; 7 Department of Gastroenterology, Hepatology and Clinical Nutrition, University Hospital Dubrava, Zagreb, 10000 Croatia; 8 Ultrasound Department, Chinese PLA General Hospital, Beijing, 100853 China; 9 Department of Ultrasound, Zhongshan Hospital Affiliated to Fudan University, Shanghai, 200032 China; 10 Department of Ultrasound, Xiamen Hospital of Traditional Chinese Medicine, Xiamen, 361009 Fujian China; 11 Department of Ultrasound, Nanjing University Medical School Affiliated Nanjing Drum Tower Hospital, Nanjing, 210008 Jiangsu China; 12 Department of Ultrasound, National Clinical Research Center for Infectious Disease, Department of Ultrasound, Shenzhen Third People’s Hospital, Second Hospital, Affiliated to Southern University of Science and Technology, Shenzhen, 518112 Guangdong China; 13 Department of Ultrasound, School of Medicine, Ruijin Hospital, Shanghai Jiaotong University, Shanghai, 200025 China; 14 Department of Ultrasound, Guangzhou Eighth People’s Hospital, Guangzhou Medical University, Guangzhou, 510600 Guangdong China; 15 Department of Abdominal Ultrasound, the First Affiliated Hospital of Xinjiang Medical University, Urumqi, 830054 Xinjiang Uygur Autonomous Region, China; 16 Department of Ultrasound, Beijing You’an Hospital Affiliated to Capital Medical University, Beijing, 100069 China; 17 Department of Ultrasound, Beijing Ditan Hospital, Capital Medical University, Beijing, 100015 China; 18 Department of Ultrasound, Ningbo Yinzhou No. 2 Hospital, Ningbo, 315100 Zhejiang China; 19 Department of Ultrasound, the First Hospital of Lanzhou University, Lanzhou, 730000 Gansu China; 20 Department of Radiology, Medical School, Zhongda Hospital, Southeast University, Nanjing, 210009 Jiangsu China

**Keywords:** Deep learning, Elastography, International multicenter study, Compensated advanced chronic liver disease

## Abstract

Accurate, noninvasive diagnosis of compensated advanced chronic liver disease (cACLD) is essential for effective clinical management but remains challenging. This study aimed to develop a deep learning-based radiomics model using international multicenter data and to evaluate its performance by comparing it to the two-dimensional shear wave elastography (2D-SWE) cut-off method covering multiple countries or regions, etiologies, and ultrasound device manufacturers. This retrospective study included 1937 adult patients with chronic liver disease due to hepatitis B, hepatitis C, or metabolic dysfunction-associated steatotic liver disease. All patients underwent 2D-SWE imaging and liver biopsy at 17 centers across China, Japan, and Europe using devices from three manufacturers (SuperSonic Imagine, General Electric, and Mindray). The proposed generalized deep learning radiomics of elastography model integrated both elastographic images and liver stiffness measurements and was trained and tested on stratified internal and external datasets. A total of 1937 patients with 9472 2D-SWE images were included in the statistical analysis. Compared to 2D-SWE, the model achieved a higher area under the receiver operating characteristic curve (AUC) (0.89 *vs* 0.83, *P* = 0.025). It also achieved a highly consistent diagnosis across all subanalyses (*P* values: 0.21–0.91), whereas 2D-SWE exhibited different AUCs in the country or region (*P* < 0.001) and etiology (*P* = 0.005) subanalyses but not in the manufacturer subanalysis (*P* = 0.24). The model demonstrated more accurate and robust performance in noninvasive cACLD diagnosis than 2D-SWE across different countries or regions, etiologies, and manufacturers.

**Supplementary Information:**

The online version contains supplementary material available at 10.1186/s42492-025-00199-6.

## Introduction

Compensated advanced chronic liver disease (cACLD) was first proposed by the Baveno VI consensus to describe the spectrum of advanced fibrosis and cirrhosis in asymptomatic patients [1]. Early and accurate discrimination of cACLD can maximally improve treatment outcomes [2, 3]. This makes the noninvasive diagnosis of cACLD vital in clinical practice [4].

Liver stiffness measurements (LSMs) obtained by two-dimensional shear wave elastography (2D-SWE) have been recommended by guidelines [5] but with inconsistent cut-off values and unstable accuracies in various studies [6–10]. For example, a meta-analysis of 53 studies showed cut-offs ranging from 7.1 to 14.1 kPa for assessing cACLD in patients with non-alcoholic fatty liver diseases [11]. Moreover, as manufacturers launched 2D-SWE systems individually, intersystem variability ranged up to 12% [12]. Guidelines point out that the LSM cut-off should be system and etiology specific [13–15], which severely limits the reliability and generalizability of 2D-SWE for identifying cACLD. Currently, when a manufacturer wants to release a new 2D-SWE system in a country or region, a multicenter study of cACLD diagnosis is required to obtain specific LSM cut-offs for different etiologies, which may be unreliable in other countries and regions [15–17]. Therefore, this expensive, time-consuming, laborious, and patient-invasive (for obtaining pathological results) process must be performed repeatedly.

Previously, a convolutional neural network (CNN)-based radiomics technique using 2D-SWE images, named deep learning radiomics of elastography (original DLRE model) was developed and its performance in staging advanced fibrosis (≥ F3) and cirrhosis (F4) evaluated in a multicenter prospective study [18]. This model was designed to automatically and quantitatively extract high-throughput image features from 2D-SWE, enabling the learning of more comprehensive disease characteristics compared to a single LSM acquisition. However, the original model had relatively inaccurate discrimination of clinically significant fibrosis (≥ F2); an updated CNN model (refined DLRE model) successfully overcame the major defect of the original version [19]. Other studies used similar methods to confirm these findings [20–22].

Both model versions were trained using 2D-SWE images acquired from patients with chronic hepatitis B (CHB) in China solely using the SuperSonic Imagine (SSI) system. From the available information, to date, there have been no reports of deep learning radiomics models whose performance has been evaluated using 2D-SWE images acquired from multi-manufacturer systems and patients with multi-etiology liver disease in multiple countries and regions worldwide.

Therefore, a generalized deep learning radiomics of elastography (DLRE-X) model was developed. Its architecture was specifically designed to eliminate noise features from different countries or regions, etiologies, and US device manufacturers in 2D-SWE images while retaining the key image features (image biomarkers) derived from crucial pathological knowledge of cACLD. To train and evaluate this updated model for cACLD identification, an international multicenter dataset of patients with chronic liver disease (CLD) was constructed. This dataset consisted of 2D-SWE images obtained from three countries and region (China, Japan, and Europe), three etiologies (CHB, chronic hepatitis C [CHC], and metabolic dysfunction-associated steatotic liver disease [MASLD]), and three manufacturers (SSI, General Electric (GE), and Mindray).

Thus, this study aimed to develop a deep learning-based radiomics model using international multicenter data and evaluate its accuracy for diagnosing cACLD compared with that of the 2D-SWE cut-off method and its robustness covering multiple countries or regions, etiologies, and US device manufacturers.

## Methods

This international multicenter study was performed in accordance with the Declaration of Helsinki and adhered to the Transparent Reporting of a Multivariable Prediction Model for Individual Prognosis or Diagnosis, or TRI-POD, statement. The study protocol was approved by the Ethics Committee of the principal investigator’s (PI) hospital (No. [2021] 02–529), and verbal informed consent was obtained.

### Study design and datasets

This study analyzed CLD with three etiologies (CHB, CHC, and MASLD) in patients from 17 academic medical centers in three countries and region (China, Japan, and Europe). 2D-SWE images were acquired by three manufacturers, including SSI (SuperSonics), GE (Logic E9), and Mindray (Resona7), between January 2012 and December 2019. The inclusion criteria were as follows: (1) patients aged 18–80 years; (2) diagnosed with CHB, CHC, or MASLD; (3) underwent LSM on 2D-SWE images acquired by the SSI, GE, or Mindray system; and (4) had liver pathological results. The exclusion criteria were as follows: (1) fewer than three 2D-SWE images of acceptable quality; (2) coinfection with other liver diseases or liver transplantation; (3) received antiviral treatments within six months prior to pathological examination; and (4) unqualified pathological results, including number of liver biopsy specimens < 2, specimen length < 15 mm, and portal areas < 6.

Patients were assigned to the training, internal test, or external test sets in two steps. First, participants with three etiologies examined by three manufacturer systems from hospitals A, B, E, F, H, I, J, K, L, and M in China and hospital N in Japan (hospital names in Supplementary Table 1), accounting for approximately 80% of the total, were assigned to the training set (64%) or internal test set (16%) using simple randomization. Second, the remaining 20% from hospitals C, D, and G in China, hospital N in Japan, and hospitals O, P, and Q in Europe were allocated to the external test set to ensure complete independence. Using this method, each patient was assigned to one of three datasets and not multiple datasets.

The DLRE-X model was developed using the training set and compared to the 2D-SWE cut-off method in all three datasets. Moreover, subanalysis comparisons were performed in terms of the diagnostic accuracy and robustness of cACLD between different countries or regions, etiologies, and manufacturers in the external test set.

### 2D-SWE image acquisition and quality control

The PI center established the standard 2D-SWE measurement procedure and promoted it to participating centers since 2012, which was recommended by many guidelines in later years [13–15, 23]. Hence, all centers follow standard 2D-SWE measurement procedures [17, 24, 25], which guarantee the quality of the 2D-SWE images. Detailed methods are provided in the Supplementary Materials.

### Clinical data and pathological evaluation

Demographic data and serological results of eligible patients were collected. All patients underwent liver biopsy within 2 weeks after 2D-SWE. The degree of fibrosis (F0-1, F2, F3, and F4) was evaluated using METAVIR (patients with CHB or CHC) or Ishak (patients with MASLD). These are the two most widely accepted scoring systems for the assessment of liver fibrosis. cACLD was defined as ≥ F3 (METAVIR scoring systems) or ≥ S4 (Ishak scoring systems) [4, 25]. Detailed methods are provided in the Supplementary Materials.

### DLRE-X

**Fig. 1:**
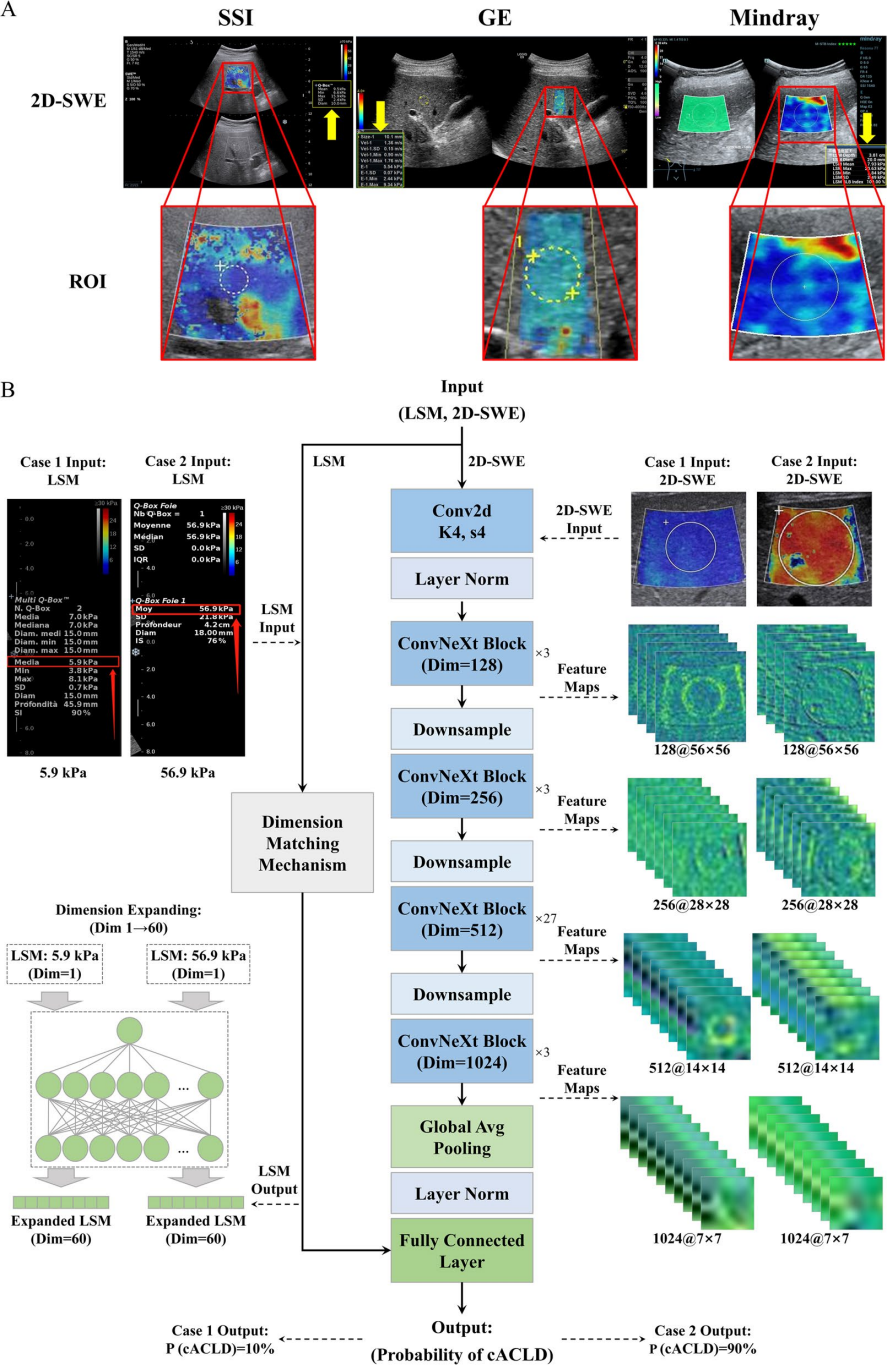
Illustration of 2D-SWE images and deep learning-based radiomics model DLRE-X architecture with a positive and a negative cACLD case. **a** Examples of 2D-SWE images obtained using three manufacturers’ systems. Red boxes are the manually selected ROIs containing the whole pseudo-color area. Yellow arrows and yellow boxes indicate the LSM values; **b** Both the LSM and 2D-SWE ROI are the inputs of DLRE-X. The middle part illustrates the structure of DLRE-X. The left side shows the dimension-matching mechanism designed for using LSM. The right side displays feature maps outputted by intermediate layers of the model. The final output gives the probability of two cases. Conv2d, two-dimensional convolutional layer, Dim, dimension

The 2D-SWE images acquired using the SSI, GE, and Mindray systems showed different appearances, colors, and manual or machine annotations in the measurement areas (Fig. 1a and Supplementary Fig. 1). The model integrated two inputs. The first was the pseudo-color area of a 2D-SWE image, which was semi-automatically cropped as the region of interest (ROI) by a US radiologist (X.L.) with more than 5 years of 2D-SWE experience, who was blinded to the pathological results, using a self-developed algorithm (Fig. 1a, red boxes; the detailed methods are shown in Supplementary Materials). The second input was the textual information of the LSM values (Fig. 1a, yellow arrows).

The model adopted the convolutional network for next generation (ConvNeXt) architecture to extract phenotypic features from 2D-SWE ROIs (Fig. 1b and Supplementary Fig. 2) [26]. Various techniques were employed, including modernizing a standard ResNet, redesigning its macro design, adopting the idea of inception, using the inverted bottleneck, increasing the kernel size, and applying different micro design choices to achieve high performance while using fewer parameters and requiring fewer computational resources [27–30]. Moreover, a transfer learning strategy was employed using pre-trained weights provided by Pytorch’s official ImageNet model [31–34]. The parameters were then fine-tuned using the training set data [35]. Offline image augmentation was performed, including random cropping, rotation, flipping, affine transformation, normalization, and tensor conversion, to improve the fine-tuning efficiency and mitigate overfitting. Owing to the dual-input design, dimension matching [36] was performed between these two inputs, and their prior weights were adjusted before dual-input feature fusion to ensure that their feature dimensions were of similar magnitudes (Fig. 1b).

All model parameters were determined using the training and internal test sets. The external test set was used to independently evaluate its performance. The performances of the single-input (ROI) and dual-input of the model were compared in all datasets and subanalyses. Detailed information on the model is shown in Supplementary Materials, and the codes of the ROI selection algorithm and the model are available at https://github.com/samadhi-fire/DLRE-3.0.

### Statistical analysis

Continuous variables were summarized as means ± SDs, and categorical variables were categorized as numbers and percentages. The area under the receiver operating characteristic (ROC) curve (AUC) was calculated to assess the diagnostic performance of the model and 2D-SWE. The prevalence, sensitivity, specificity, positive and negative predictive values, and positive and negative likelihood ratio (LR + and LR-) of maximizing the Youden index on the estimated ROCs were calculated with 95%CIs. AUCs with 95%CIs of the model and 2D-SWE were compared in all three datasets and in each subanalysis under the external test set using the Delong test. *P* < 0.05 indicated statistical significance. Statistical analysis was performed using SPSS software for Windows, V.20.0 (SPSS).

## Results

### Baseline dataset characteristics

**Fig. 2:**
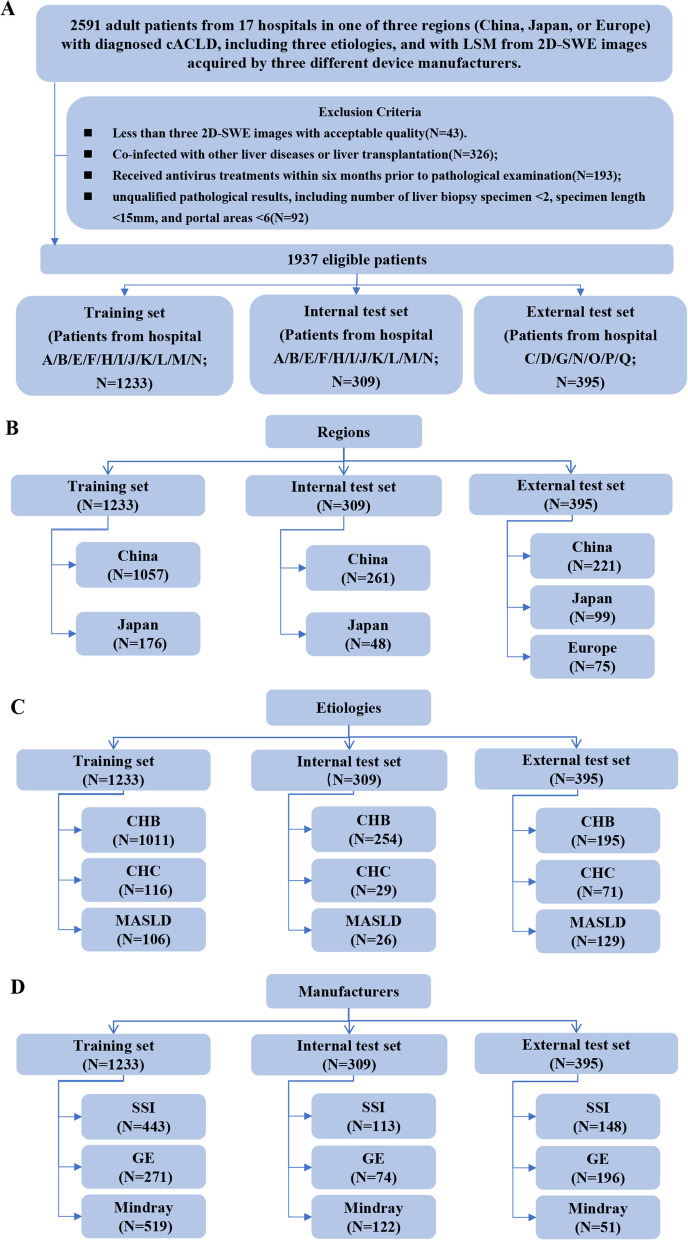
Flowchart of patient enrollment process for the training, internal test, and external test sets. (**a**) The procedure of patient enrollment. Of 2591 patients, 654 were excluded and 1937 patients with 9472 2D-SWE images were selected for analysis. Distribution of the (**b**) country or region, (**c**) etiology, and (**d**) manufacturer subanalyses

Of 2591 patients, 654 were excluded because of insufficient number of images, co-infection or liver transplantation, receiving antiviral treatments before liver biopsy, and unquantified histological results, and 1937 patients with 9472 2D-SWE images were selected for analysis after exclusion (Fig. 2a). Among them, 1233 patients with 6150 images, 309 patients with 1543 images, and 395 patients with 1779 images were allocated to the training, internal, or external test sets, respectively (Supplementary Materials). The detailed distribution of the included patients regarding the country or region, etiologies, manufacturers, and participating centers is shown in Figs. 2b-d and Supplementary Table 1. Table 1 lists the baseline characteristics. There was no evidence of differences among the training, internal, and external test sets (all *P* > 0.05).

**Table 1:** Baseline characteristics of the training, internal test, and external test sets

Characteristics	Training	Internal test set	External test set	*P* value
No. of patients	1233	309	395	0.54
Age (year)*	58.92 ± 15.63	60.62 ± 12.83	60.85 ± 13.86	0.57
Age range (year)	19–79	21–71	19–79	0.57
Sex (M)	653 (53%)	159 (52%)	216 (55%)	0.54
Sex (F)	580 (47%)	150 (48%)	179 (45%)	0.54
BMI*	25.70 ± 4.87	27.07 ± 5.67	25.45 ± 5.73	0.25
AST (U/L)*	51.43 ± 50.86	47.87 ± 31.35	45.56 ± 28.23	0.57
ALT (U/L)*	62.07 ± 74.97	51.87 ± 34.26	58.57 ± 54.45	0.66
ALB (g/L)*	4.08 ± 0.50	4.10 ± 0.23	4.12 ± 0.57	0.76
TB (umol/L)*	0.73 ± 0.40	0.71 ± 0.29	0.80 ± 0.38	0.31
DB (umol/L)*	0.43 ± 0.22	0.42 ± 0.18	0.48 ± 0.21	0.25
IB (umol/L)*	0.29 ± 0.18	0.27 ± 0.13	0.31 ± 0.17	0.43
GGT (U/L)*	61.66 ± 69.67	57.65 ± 53.52	69.80 ± 119.86	0.70
PT (s)*	12.92 ± 2.09	12.70 ± 1.29	12.96 ± 2.52	0.82
Fibrosis degree				
F0-1	460 (38%)	127 (41%)	168 (43%)	0.51
F2	349 (28%)	69 (22%)	84 (21%)	0.61
F3	210 (17%)	60 (19%)	59 (15%)	0.53
F4	214 (17%)	53 (17%)	84 (21%)	0.51
cACLD or not				
non-cACLD	809 (66%)	196 (63%)	252 (64%)	0.55
cACLD	424 (34%)	113 (37%)	143 (36%)	0.53

Data are numbers of participants, with percentages in parentheses, for categorical variables

*AST* aspartate aminotransferase, *BMI* body mass index, calculated as weight in kilograms divided by height in meters squared, *ALT* alanine aminotransferase, *ALB* albumin, *TB* total bilirubin, *DB* direct bilirubin, *IB* indirect bilirubin, *GGT* gamma-glutamyl transpeptidase, *PT* prothrombin activity, *F0-1* no or minor fibrosis, *F2* clinically significant fibrosis, *F3* advanced fibrosis, *F4* cirrhosis

*Data are means ± SDs for continuous variables. *P* values were calculated between the training, internal test, and external test sets using analysis of variance (ANOVA) for continuous variables and Fisher’s exact test for categorical variables

### Overall diagnostic performance comparisons between DLRE-X and 2D-SWE

**Fig. 3:**
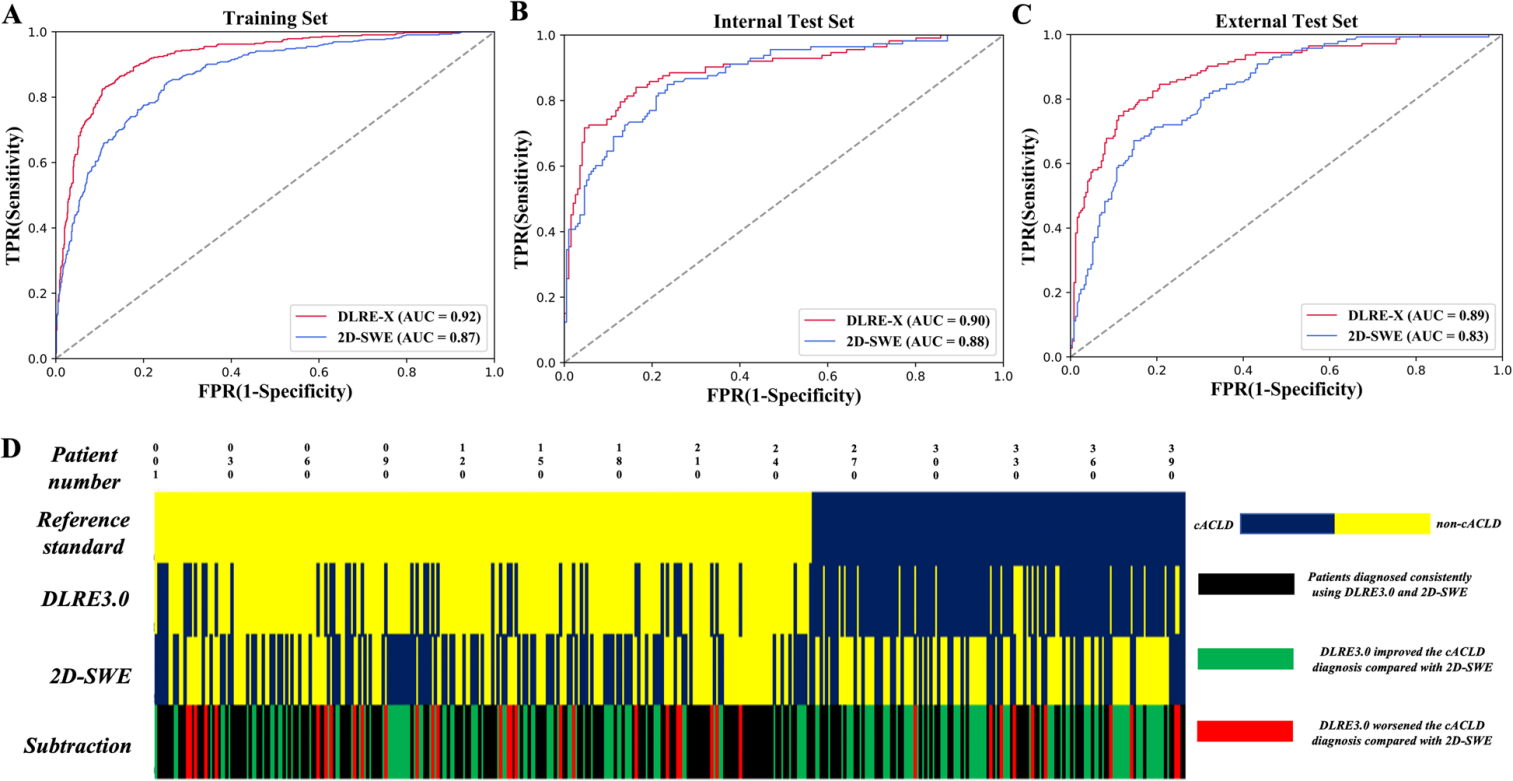
Overall comparisons between the deep learning-based radiomics model DLRE-X and 2D-SWE. ROC curves of DLRE-X and 2D-SWE in the training (**a**), internal test (**b**), and external test sets (**c**). Heat plot of DLRE-X and 2D-SWE in the external test set (**d**)

Figure 3a shows the overall diagnostic performances of the model and 2D-SWE in the training, internal test, and external test sets for diagnosing cACLD. The hyperparameters of the model and the LSM cut-off values of 2D-SWE are shown in Supplementary Table 2. In the training set, the model achieved higher AUCs (0.92; 95%CI: 0.91, 0.94 *vs* 0.87; 95%CI: 0.85, 0.89; *P* < 0.001) than 2D-SWE. The sensitivity, specificity, and other quantitative indices are listed in Table 2 and Supplementary Table 3. However, in the internal test set, no evidence of a difference was found between the model and 2D-SWE (AUC: 0.90; 95%CI: 0.86, 0.93 *vs* 0.88; 95%CI: 0.83, 0.91; *P* = 0.49).

**Table 2:** Diagnostic performances of the deep learning-based radiomics model (DLRE-X) and 2D-SWE in the training, internal test, and external test sets

	**N**	**Prevalence (%)**	**Sensitivity (%)**	**Specificity(%)**	**AUC**	***P* value**
**Training set**						
The model	1233	34.3 [424/1233] (31.7, 37.0)	87 [370/424] (85, 90)	84 [678/809] (81, 86)	0.92 (0.91, 0.94)	0.0002
2D-SWE	1233	34.3 [424/1233] (31.7, 37.0)	84 [358/424] (81, 88)	75 [598/799] (72, 78)	0.87 (0.85, 0.89)	
**Internal test set**						
The model	309	36.6 [113/309] (31.2, 42.0)	81 [91/113] (74, 86)	86 [168/196] (81, 90)	0.90 (0.86, 0.93)	0.49
2D-SWE	309	36.6 [113/309] (31.2, 42.0)	77 [87/113] (71, 83)	67 [132/196] (59, 75)	0.88 (0.83, 0.91)	
**External test set**						
The model	395	36.2 [143/395] (31.4, 41.0)	83 [118/143] (77, 87)	81 [203/252] (76, 84)	0.89 (0.86, 0.91)	0.025
2D-SWE	395	36.2 [143/395] (31.4, 41.0)	72 [103/143] (64, 79)	75 [189/252] (69, 80)	0.83 (0.79, 0.87)	
**Subanalysis of external test set**						
** Country or region-China**						
The model	221	40.2 [89/221] (33.8, 46.8)	82 [73/89] (75, 88)	79 [104/132] (73, 84)	0.87 (0.83, 0.91)	0.003
2D-SWE	221	40.2 [89/221] (33.8, 46.8)	64 [57/89] (53, 74)	69 [91/132] (60, 77)	0.75 (0.69, 0.81)	
** Country or region-Japan**						
The model	99	36.4 [36/99] (26.7, 46.0)	83 [30/36] (72, 93)	83 [52/63] (74, 90)	0.91 (0.85, 0.95)	> 0.99
2D-SWE	99	36.4 [36/99] (26.7, 46.0)	86 [31/36] (71, 95)	75 [47/63] (62, 85)	0.91 (0.83, 0.96)	
** Country or region-Europe**						
The model	75	24.0 [18/75] (14.1, 33.9)	83 [15/18] (67, 96)	82 [47/57] (74, 91)	0.91 (0.84, 0.97)	0.69
2D-SWE	75	24.0 [18/75] (14.1, 33.9)	83 [15/18] (59, 96)	90 [51/57] (79, 96)	0.93 (0.85, 0.98)	
** Etiology-CHB**						
The model	195	44.1 [86/195] (37.1, 51.1)	83 [71/86] (76, 89)	78 [85/109] (72, 85)	0.87 (0.82, 0.91)	0.004
2D-SWE	195	44.1 [86/195] (37.1, 51.1)	64 [55/86] (53, 74)	68 [74/109] (58, 77)	0.75 (0.68, 0.81)	
** Etiology-CHC**						
The model	71	47.9 [34/71] (36.0, 59.8)	85 [29/34] (75, 94)	65 [24/37] (51, 77)	0.86 (0.78, 0.93)	0.77
2D-SWE	71	47.9 [34/71] (36.0, 59.8)	82 [28/34] (66, 93)	70 [26/37] (53, 84)	0.84 (0.74, 0.92)	
** Etiology-MASLD**						
The model	129	17.8 [23/129] (11.1, 24.5)	78 [18/23] (64, 92)	89 [94/106] (83, 93)	0.90 (0.82, 0.96)	0.86
2D-SWE	129	17.8 [23/129] (11.1, 24.5)	87 [20/23] (77, 90)	84 [89/106] (87, 97)	0.91 (0.85, 0.95)	
** Manufacturer-SSI**						
The model	148	21.6 [32/148] (14.9, 28.3)	69 [22/32] (54, 81)	81 [94/116] (75, 87)	0.84 (0.78, 0.90)	0.77
2D-SWE	148	21.6 [32/148] (14.9, 28.3)	78 [25/32] (60, 91)	78 [90/116] (69, 85)	0.87 (0.80, 0.92)	
** Manufacturer-GE**						
The model	196	50.5 [99/196] (43.4, 47.6)	87 [86/99] (81, 92)	76 [74/97] (69, 83)	0.90 (0.86, 0.93)	0.94
2D-SWE	196	50.5 [99/196] (43.4, 47.6)	69 [68/99] (58, 78)	80 [78/97] (71, 88)	0.87 (0.81, 0.91)	
** Manufacturer-Mindray**						
The model	51	23.5 [12/51] (11.5, 35.6)	83 [10/12] (64, 100)	90 [35/39] (81, 97)	0.88 (0.76, 0.99)	0.65
2D-SWE	51	23.5 [12/51] (11.5, 35.6)	83 [10/12] (52, 98)	56 [22/39] (40, 72)	0.81 (0.67, 0.90)	

Data in parentheses are 95%CIs, and data in brackets are numbers of patients. The AUC of the model was statistically compared with the AUC of 2D-SWE in each dataset or subanalysis using the Delong test

In the external test sets, the model achieved a higher AUC (0.89; 95%CI: 0.86, 0.91) than 2D-SWE (0.83; 95%CI: 0.79, 0.87) (*P* = 0.02). It achieved better diagnostic performance for most quantitative indices (Table 2 and Supplementary Table 3). Figure 3d shows the results of both methods for each patient in the external set, demonstrating that the model provided fewer false negatives (blue stripes in yellow areas, 49 *vs* 114, *P* < 0.001) and fewer false positives (yellow stripes in blue areas, 25 *vs* 90, *P* < 0.001) than 2D-SWE. Some failure cases of the model are presented in Supplementary Fig. 3, revealing that misdiagnosed cases were similarly to correct cases.

The net benefits of the two methods in the decision curve analysis are shown in Supplementary Fig. 4. The area under the red decision curve (the model) exceeds the blue area (2D-SWE) in all three datasets.

### Single- *vs* dual-input performance of DLRE-X

The results of the single- and dual-input evaluations are shown in Supplementary results and Supplementary Table 4. Dual-input provided a better AUC than single-input in the external test set (0.89; 95%CI: 0.86, 0.91 *vs* 0.83; 95%CI: 0.79, 0.86; *P* = 0.02).

### Diagnostic accuracy and robustness comparisons between DLRE-X and 2D-SWE among different subanalyses

#### Country or region

**Fig. 4:**
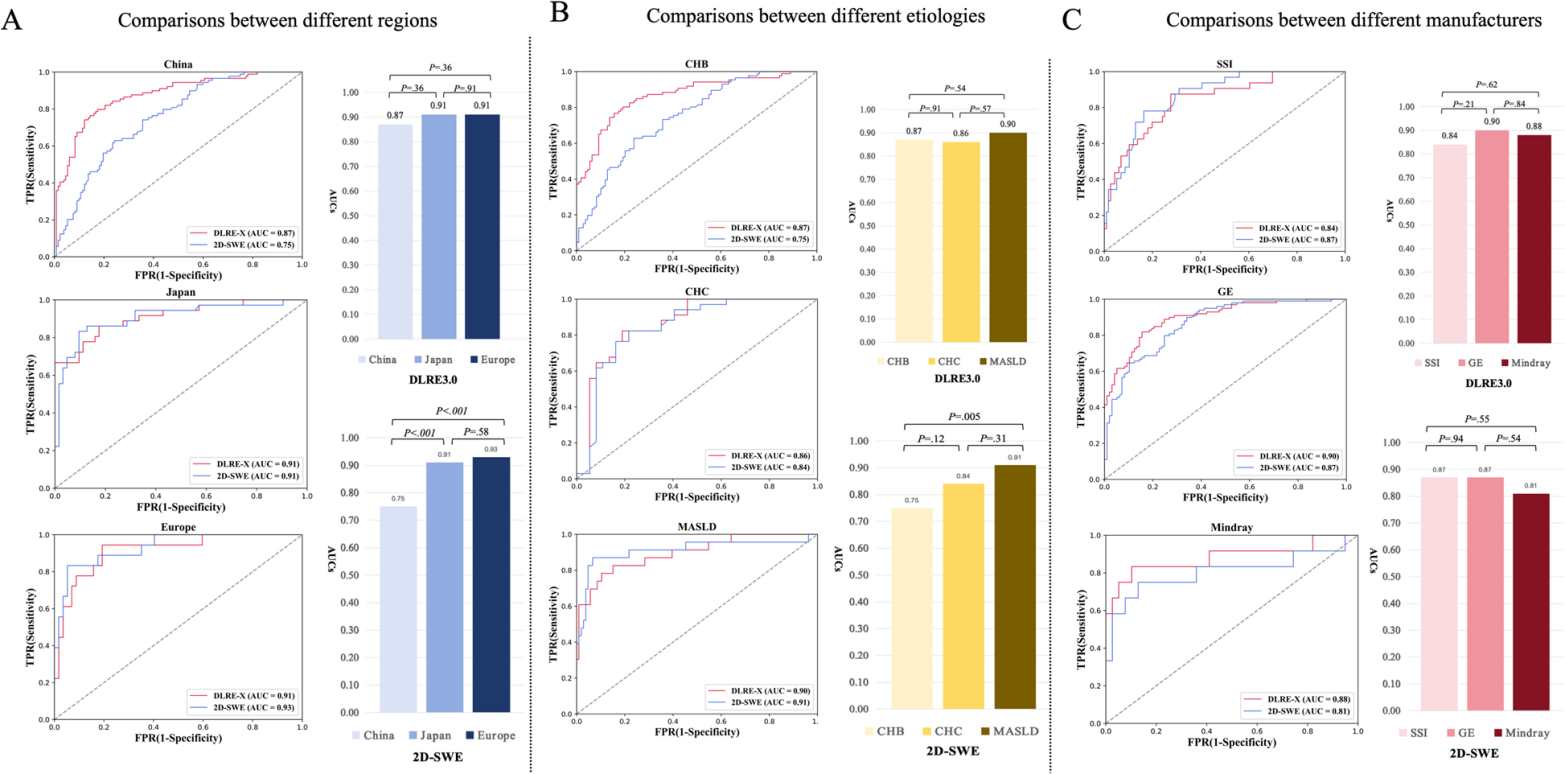
Subanalysis comparisons between DLRE-X and 2D-SWE under the external test set. **a** ROC curves and AUC values of DLRE-X and 2D-SWE for different country or region (Chinese, Japanese, and European subanalyses); **b** ROC curves and AUC values of DLRE-X and 2D-SWE for different etiologies (CHB, CHC, and MASLD subanalyses); **c** ROC curves and AUC values of DLRE-X and 2D-SWE for different manufacturers (SSI, GE, and Mindray subanalyses). AUC values of each method within each subanalysis were statistically compared pairwise using the Delong test. DLRE-X achieved highly consistent diagnosis across all subanalyses (*P* values: 0.21–0.91), whereas 2D-SWE exhibited different AUCs in country and region (*P* < 0.001) and etiology (*P* = 0.005) subanalyses, but not in the manufacturer subanalysis (*P* = 0.24)

Figure 4a and Supplementary Table 5 show the ROC curves and quantitative indices of the model and 2D-SWE for the three countries and region (China, Japan, and Europe) under the external test set. In the Chinese subanalysis, the model had better AUC values than 2D-SWE (0.87; 95%CI: 0.83, 0.91 *vs* 0.75; 95%CI: 0.69, 0.81; *P* = 0.003), but in the Japanese and European subanalyses, there was no evidence of differences between the model and 2D-SWE (Japan: 0.91; 95%CI: 0.85, 0.95 *vs* 0.91; 95%CI: 0.83, 0.96; *P* > 0.99; Europe: 0.91; 95%CI: 0.84, 0.97 *vs* 0.93; 95%CI: 0.85, 0.98; *P* = 0.69).

Diagnostic robustness comparisons revealed different consistencies between the two methods in different countries and regions. Using the model to diagnose cACLD in different countries and regions, no differences were found between the AUCs (Fig. 4a, upper bar chart, AUC range: 0.87, 0.91; *P* = 0.21). However, 2D-SWE exhibited different AUCs between the Chinese, Japanese, and European subanalyses (Fig. 4a, lower bar chart, AUC range: 0.75, 0.93; *P* < 0.001).

#### Etiologies

The model provided a better AUC than 2D-SWE in the CHB subanalysis (0.87; 95%CI: 0.82, 0.91 *vs* 0.75; 95%CI: 0.68, 0.81; *P* = 0.004) (Fig. 4b and Supplementary Table 6). However, in both the CHB and MASLD subanalyses, there were differences between AUCs for the model and 2D-SWE (CHC: 0.86; 95%CI: 0.78, 0.93 *vs* 0.84; 95%CI: 0.74, 0.92; *P* = 0.77; MASLD: 0.90; 95%CI: 0.82, 0.96 *vs* 0.91; 95%CI: 0.85, 0.95; *P* = 0.86).

The diagnostic robustness comparisons showed no evidence of differences between AUCs for the model across different etiologies (Fig. 4b, upper bar chart, AUC range: 0.87, 0.90; *P* = 0.27), but 2D-SWE exhibited different AUCs between them (Fig. 4b, lower bar chart, AUC range: 0.75, 0.91; *P* = 0.005).

#### US device manufacturers

When comparing AUC values between the model and 2D-SWE in all three manufacturer subanalyses, there was no evidence of statistical differences: SSI (0.84; 95%CI: 0.78, 0.90 *vs* 0.87; 95%CI: 0.80, 0.92; *P* = 0.77), GE (0.90; 95%CI: 0.86, 0.93 *vs* 0.87; 95%CI: 0.81, 0.91; *P* = 0.94), Mindray (0.88; 95%CI: 0.76, 0.99 *vs* 0.81; 95%CI: 0.67, 0.90; *P* = 0.65) (Fig. 4c, Table 2, and Supplementary Table 7).

Diagnostic robustness comparisons showed no evidence of differences between the AUCs for either the model or 2D-SWE across different manufacturers (Fig. 4c, upper bar chart, AUC range: 0.84, 0.90; *P* = 0.55; lower bar chart, AUC range: 0.81, 0.87; *P* = 0.24).

#### Single- *vs* dual-input performance of DLRE-X in subanalyses

The results of the single- *vs* dual-input of the model in the country or region, etiology, and manufacturer subanalyses are presented in Supplementary Tables 8–10. Dual-input provided higher AUCs than single-input in the Chinese (0.87; 95%CI: 0.83, 0.91; *vs* 0.78; 95%CI: 0.73, 0.84; *P* = 0.03) and CHB (0.87; 95%CI: 0.82, 0.91; *vs* 0.77; 95%CI: 0.72, 0.83; *P* = 0.02) subanalyses, but there was no evidence of differences in the other subanalyses (all *P* > 0.05).

#### Poor robustness of 2D-SWE in cACLD discrimination

The poor robustness of 2D-SWE in cACLD discrimination was further demonstrated by listing the optimal LSM cut-offs for each subanalysis under the external test set (Supplementary Table 11). There was a 33.5% [(10.66–7.09)/10.66] difference between the lowest cut-off (7.09 kPa, GE subanalysis) and the largest cut-off (10.66 kPa, Mindray subanalysis).

## Discussion

Noninvasive and accurate diagnosis of cACLD is necessary to optimize treatment outcomes. However, the lack of a universal cut-off for 2D-SWE limits its reliability and generalizability in identifying cACLD. Thus, the accuracy and robustness of DLRE-X and 2D-SWE for noninvasive diagnosis of cACLD were investigated. A total of 1937 patients with CLD who underwent 2D-SWE and liver biopsy at 17 centers in China, Japan, and Europe were included. Their data were used to develop and evaluate the DLRE-X model as well as to make comparisons with 2D-SWE. The results demonstrate that the model achieved significantly better diagnostic accuracy in the external test set. More importantly, when the robustness of identifying cACLD was compared using these two methods across different subanalyses, DLRE-X was found to achieve highly reproducible AUCs, whereas 2D-SWE exhibited significantly different AUCs in patients from different countries and regions or with different etiologies.

Although a considerable number of studies have assessed the effectiveness of using radiomics models to stage liver fibrosis [18–22], there are no real international data covering multiple manufacturers and etiologies to evaluate their potential impact on the performance of radiomics models. Durot et al. [37] previously proposed a machine learning-based model capable of analyzing point SWE and 2D-SWE images from two manufacturers (Siemens and Philips) to grade significant fibrosis (≥ F2) in patients with CLD of multiple etiologies. However, this was a single-center retrospective study with only 232 patients, using magnetic resonance elastography as the reference standard instead of pathological results. Therefore, this international multicenter study offers solid evidence that eliminates doubts regarding the generalizability of radiomics models.

Of note, the data of European patients were not included in the training and internal test sets but were only used for the independent external test set. One important reason for this was the relatively small sample size of European patients, which made it difficult to effectively support training, internal testing, and external testing simultaneously. Another reason was that such a design could verify whether DLRE-X could achieve an accurate and consistent diagnosis in European patients in the absence of European training data. The results showed that this was the case, suggesting that the model may be applicable to other regions/countries even if patients in those places were not involved in the model training process.

In addition, in the external test set, the AUC of the model was significantly better than that of 2D-SWE only in the Chinese subanalysis (0.87 *vs* 0.75, *P* = 0.003). No evidence of significant differences was observed in the European or Japanese subanalyses (both *P* > 0.05). In addition, AUCs of 2D-SWE in China were much lower than that in Europe and Japan (China *vs* Europe *vs* Japan: 0.75 *vs* 0.93 *vs* 0.91, *P* < 0.001). This was probably because the Chinese subanalysis was subject to more confounding factors. It included 221 patients from three hospitals examined using US devices from three manufacturers, whereas the Japanese and European subanalyses included only 99 patients from one hospital using GE systems and 75 patients from three hospitals using SSI systems, respectively. When including a larger sample size and more hospitals and manufacturers and the tested subanalyses were closer to the real world, the performance of 2D-SWE worsened, and the advantage of the model became more obvious, as shown in the Chinese subanalysis comparison. Such limitations of the LSM cut-offs are consistent with those reported in previous studies. A multicenter study by Degos et al. [38] showed a lower AUC than a single-center study by Cardoso et al. [39] (number of patients: 284 *vs* 202; AUC: 0.78 *vs* 0.87), even though both studies were performed in the same region using the same manufacturer.

Many other studies have also indicated that the adoption of LSM cut-offs for diagnosing cACLD is severely affected by various factors, including country and region, manufacturer, etiology, inflammation, and steatosis, yielding a wide range of thresholds with varied performances [5–9]. Thus, the worldwide promotion of this technique suffers from the lack of a universally reliable cut-off value [40, 41]. There are more than 1600 Grade IIIA hospitals in China that are responsible for managing more than 440 million patients with CLD in their routine clinical practice [42, 43], but they use US elastography devices from different manufacturers. Several multicenter clinical trials have been conducted to determine optimal LSM cut-offs for different manufacturers [44, 45]. However, these tremendous efforts have already been made in many other countries and regions and will probably have to be made again when new US devices are released. The current study demonstrates that the DLRE-X is an effective approach for solving this challenge. The model works for patients with any etiology of CLD or for any 2D-SWE systems in any country. Once it is trained with sufficient data, ultrasound radiologists only need to perform a standardized manual selection of the ROI in the daily workflow to obtain an accurate and reliable diagnosis of cACLD.

The biggest challenge of this study was the design and development of DLRE-X because 2D-SWE images are remarkably different from different manufacturers. To establish this model, a state-of-the-art ConvNeXt [28], dual-input (LSM and 2D-SWE ROI) design, transfer learning strategy [32], and ROI selection and data argumentation protocol inherited from the original and refined DLRE models were adopted [18, 19]. The impacts of single and dual inputs on the performance of the model were also compared, which proved that dual-input improved the overall diagnostic accuracy. Therefore, the integration of LSM and pseudo-color image for deep learning analysis is necessary and should be widely used in future clinical applications. The overall performance of the model will continue to improve as more data are added to the training. Such a self-evolution capability is not available in the conventional 2D-SWE method. Another important point is that diagnostic inconsistencies across different devices, etiologies, and regions are not exclusive to 2D-SWE; they also appear in other imaging modalities (such as magnetic resonance elastography). The DLRE-X model offers methodological viability for constructing artificial intelligence models that can be applied across devices, etiologies, and regions and may help other artificial intelligence modalities.

This study had several limitations. First, it was a retrospective study. Second, there were prevalence biases. Owing to the different disease prevalence in different regions, most patients with CHB were from China, whereas all European patients had MASLD. The ALT levels (U/L) were higher in this study than that in other studies (current study *vs* Zheng et al. [17]: 60.0; 95%CI: 56.2, 63.7; *vs* 43.0; 95%CI: 26.0, 87.5; *P* = 0.01) [25], which was probably a confounding factor affecting the performance of 2D-SWE. Third, the sample size was relatively small for countries/regions other than China. Finally, only three regions, three manufacturers, and three etiologies were included. A larger sample size, including more regions/manufacturers and etiologies, is required in future studies.

In summary, a deep learning-based radiomics model achieved more accurate and robust performance in the noninvasive diagnosis of cACLD than 2D-SWE across different countries and regions, manufacturers, and etiologies. Future evaluations of this model should be based on the current study by evaluating more etiologies, US device manufacturers, countries and regions, and countries.

## Conclusions

The model achieved more accurate and robust performance in the noninvasive diagnosis of cACLD than 2D-SWE across different countries and regions, etiologies, and manufacturers.

## Supplementary Information

Supplementary Material 1.

Supplementary Material 2.

Supplementary Material 3.

## Data Availability

The 2D-SWE images and datasets generated during or analyzed in this study are not publicly available due to restrictions by privacy laws. The 2D-SWE images, LSM values and related clinical datasets are held by the Department of Ultrasound, Guangdong Key Laboratory of Liver Disease Research, Third Affiliated Hospital of Sun Yat-Sen University, Guangzhou, China. Requests for sharing of all data and material should be addressed to the corresponding author within 15 years of the date of publication of this article and include a scientific proposal. The datasets will be shared after the approval of Prof. Ping Liang with a signed data access agreements.

## Abbreviations

cACLD: Compensated advanced chronic liver disease
LSM: Liver stiffness measurement
CNN: Convolutional neural network
CHB: Chronic hepatitis B
CHC: Chronic hepatitis C
CLD: Chronic liver disease
MASLD: Metabolic dysfunction-associated steatotic liver disease
ROC: Receiver operating characteristic
AUC: Area under receiver characteristics curve
2D-SWE: Two-dimensional shear wave elastography
SSI: SuperSonic Imagine
GE: General Electric
DLRE-X: Generalized deep learning radiomics of elastography
ROI: Region of interest
Conv2d: Two-dimensional convolutional layer
Dim: Dimension
ConvNeXt: Convolutional network for next generation
AST: Aspartate aminotransferase
BMI: Body mass index
ALT: Alanine aminotransferase
ALB: Albumin
TB: Total bilirubin
DB: Direct bilirubin
IB: Indirect bilirubin
GGT: Gamma-glutamyl transpeptidase
PT: Prothrombin activity
F0-1: No or minor fibrosis
F2: Clinically significant fibrosis
F3: Advanced fibrosis
F4: Cirrhosis
