# Supplementary material for: Deep learning radiomics of elastography for diagnosing compensated advanced chronic liver disease: an international multicenter study
Source: Vis Comput Ind Biomed Art. 2025 Aug 15;8:19. doi: 10.1186/s42492-025-00199-6 (PMC12354435; doi:10.1186/s42492-025-00199-6)
Supplement: Supplementary file 1 — Supplementary Material 1. [file 42492_2025_199_MOESM1_ESM.docx]

**Supplementary materials-Tables**

Supplementary Table 1. The Distribution of Enrolled Patients in Different Centers Examined By Different Equipment and Different Etiologies.

| Region | Code | Participation centers | Manufacturers | Etiologies | No. |
| --- | --- | --- | --- | --- | --- |
| China | A | The Third Affiated Hospital of Sun Yat-Sen University | SSI | CHB/CHC/MASLD | 532/15/9 |
|  |  |  | GE | CHB/CHC/MASLD | 107/1/13 |
|  |  |  | Mindray | CHB/CHC/MASLD | 266/1/12 |
|  | B | Chinese PLA general Hospital | Mindray | CHB | 39 |
|  | C | Zhongshan Hospital Affiliated with Fudan University | GE | CHB | 96 |
|  |  |  | SSI | CHB/MASLD | 30/19 |
|  | D | Beijing You’an Hospital, Capital Medical University | SSI | CHB/CHC/MASLD | 9/9/7 |
|  | E | Xiamen Traditional Chinese Medicine Hospital | Mindray | CHB | 96 |
|  | F | The Third People’s Hospital of Shenzhen | Mindray | CHB | 46 |
|  | G | Affiliated Drum Tower Hospital, Medical School of Nanjing University | Mindray | CHB | 51 |
|  | H | Ruijing Hospital, Shanghai Jiaotong University School of Medicine | Mindray | CHB | 48 |
|  | I | The Eighth People's Hospital of Guangzhou | Mindray | CHB/CHC | 35/11 |
|  | J | Ningbo Yinzhou NO.2 Hospital | Mindray | CHB | 26 |
|  | K | The First Affiliated Hospital of Xinjiang Medical University | Mindray | CHB | 25 |
|  | L | The First Affiliated Hospital of Lanzhou University | Mindray | CHB | 13 |
|  | M | Beijing Ditan Hospital | Mindray | CHB | 21 |
| Japan | N | Itwa Medical University | GE | CHB/CHC/MASLD | 15/170/138 |
| European | O | Catholic University of Sacred Heart | SSI | CHB/CHC/MASLD | 3/4/45 |
|  | P | Bordeaux University Hospital | SSI | CHC/MASLD | 2/12 |
|  | Q | University Hospital Dubrava | SSI | CHC/MASLD | 3/6 |

Note.­SSI, SuperSonic Imagine; GE, General Electric; CHB, chronic hepatitis B; CHC, chronic hepatitis C; MASLD, metabolic dysfunction associated steatotic liver disease.

Supplementary Table 2 Hyperparameters needs to be optimized in model training.

|  | Index | Hyperparameter | Option |
| --- | --- | --- | --- |
| 2D-SWE | 1 | Cut-offs | 8.38kPa |
| DLRE-X | 1 | Number of ConvNeXt blocks | 3, 3, 27, 3 |
|  | 2 | Number of linear layers | 2 |
|  | 3 | Dropout rate | 0.5 |
|  | 4 | Batch size | 32 |
|  | 5 | Epochs | 50 |
|  | 6 | Activation function | GELU, LeakyReLU |
|  | 7 | Initializer | Kaiming normal, normal |
|  | 8 | Optimizer | AdamW |

Note. ­2D-SWE, two-dimensional shear wave elastography; DLRE-X , deep learning radiomics of shear wave elastography version 3.0.

Supplementary Table 3. Diagnostic performances of the deep learning-based radiomics model (DLRE-X ) and 2D-SWE in the training, internal test and external test set.

|  | **PPV**  **%** | **NPV**  **%** | **LR+** | **LR-** |
| --- | --- | --- | --- | --- |
| **Training set** |  |  |  |  |
| The model | 74  (70, 77) | 93  (91, 94) | 5.39  (4.71, 6.22) | 0.15  (0.12, 0.18) |
| 2D-SWE | 64  (60, 68) | 90  (88, 92) | 3.35  (3.20, 3.50) | 0.21  (0.20, 0.30) |
| **Internal test set** |  |  |  |  |
| The model | 77  (70, 83) | 89  (85, 92) | 5.64  (4.23, 7.83) | 0.23  (0.16, 0.30) |
| 2D-SWE | 68  (59, 75) | 88  (83, 93) | 3.58  (3.20, 4.00) | 0.23  (0.10, 0.40) |
| **External test set** |  |  |  |  |
| The model | 71  (65, 76) | 89  (86, 93) | 4.24  (3.4, 5.35) | 0.22  (0.15, 0.29) |
| 2D-SWE | 62  (54, 70) | 83  (77, 87) | 2.88  (2.50, 3.30) | 0.37  (0.30, 0.50) |

Note.­Data in parentheses are 95% CIs. 2D-SWE, two-dimensional shear wave elastography; PPV, positive predictive values; NPV, negative predictive values; LR, likelihood ratio.

Supplementary Table 4.Diagnostic performances of single- and dual-input of the deep learning-based radiomics model (DLRE-X ) in training, internal test and external test sets.

|  | **N** | **Prevalence** | **AUC** | **Sensitivity**  **%** | **Specificity**  **%** | **PPV**  **%** | **NPV**  **%** | **LR+** | **LR-** |
| --- | --- | --- | --- | --- | --- | --- | --- | --- | --- |
| **Training set** | | | |  |  |  |  |  |  |
| Dual-input | 1233 | 34.4  (31.7, 37.0) | 0.92  (0.91, 0.94) | 87  (85, 90) | 84  (81, 86) | 74  (70, 77) | 93  (91, 94) | 5.39  (5.28, 5.71) | 0.15  (0.14, 0.12) |
| Single-input | 1233 | 34.4  (31.7, 37.0) | 0.94  (0.93, 0.95) | 85  (82, 88) | 89  (87, 91) | 80  (77, 83) | 92  (90, 93) | 7.76  (6.77, 8.6) | 0.16  (0.18, 0.13) |
| **Internal test set** | | | |  |  |  |  |  |  |
| Dual-input | 309 | 36.6  (31.2, 42.0) | 0.9  (0.86, 0.93) | 81  (74, 86) | 86  (81, 90) | 76  (70, 83) | 88  (85, 92) | 5.64  (5.01, 6.24) | 0.23  (0.21, 0.23) |
| Single-input | 309 | 36.6  (31.2, 42.0) | 0.88  (0.84, 0.91) | 73  (65, 80) | 87  (83, 91) | 76  (69, 82) | 85  (80, 89) | 5.47  (4.55, 5.4) | 0.32  (0.35, 0.33) |
| **External test set** | | | |  |  |  |  |  |  |
| Dual-input | 395 | 36.2  (31.4, 41.0) | 0.89  (0.86, 0.91) | 83  (77, 87) | 81  (76, 84) | 71  (64, 76) | 89  (86, 92) | 4.24  (4.73, 4.09) | 0.22  (0.21, 0.22) |
| Single-input | 395 | 36.2  (31.4, 41.0) | 0.83*  (0.79, 0.86) | 66  (60, 73) | 88  (84, 91) | 75  (69, 81) | 82  (78, 86) | 5.4  (8.07, 4.6) | 0.38  (0.37, 0.38) |

Note.­Data in parentheses are 95% CIs. AUC of the Dual- and single-input of the model were statistically compared in each dataset using the Delong test (*P < 0.05, **P < 0.01, ***P < 0.001). N, number of patients; AUC, area under the receiver characteristics curve; PPV, positive predictive value; NPV, negative predictive value; LR, likelihood ratio.

Supplementary Table 5. Diagnostic performances of the deep learning-based radiomics model (DLRE-X ) and 2D-SWE in China, Japan and Europe sub-groups under the external test set.

|  | **PPV**  **%** | **NPV**  **%** | **LR+** | **LR-** |
| --- | --- | --- | --- | --- |
| **China** |  |  |  |  |
| The model | 73  (65, 79) | 87  (82, 92) | 3.87  (3.00, 5.22) | 0.23  (0.15, 0.32) |
| 2D-SWE | 58  (48, 68) | 74  (65, 82) | 2.06  (1.70, 2.50) | 0.52  (0.40, 0.80) |
| **Japan** |  |  |  |  |
| The model | 73  (60, 85) | 90  (83, 96) | 4.77  (3.16, 8.47) | 0.2  (0.08, 0.35) |
| 2D-SWE | 66  (51, 79) | 90.  (79., 97) | 3.39  (2.80, 4.10) | 0.19  (0.07, 0.50) |
| **Europe** |  |  |  |  |
| The model | 60  (43, 76) | 94  (88, 98) | 4.75  (3.09, 8.84) | 0.2  (0.05, 0.41) |
| 2D-SWE | 71  (48, 89) | 94  (85, 99) | 7.92  (6.30, 9.90) | 0.19  (0.05, 0.70) |

Note.­Data in parentheses are 95% CIs. 2D-SWE, two-dimensional shear wave elastography; PPV, positive predictive value; NPV, negative predictive value; LR, likelihood ratio.

Supplementary Table 6. Diagnostic performances of the deep learning-based radiomics model (DLRE-X ) and 2D-SWE in CHB, CHC and MASLD subgroups under the external test set

|  | **PPV**  **%** | **NPV**  **%** | **LR+** | **LR-** |
| --- | --- | --- | --- | --- |
| **CHB** |  |  |  |  |
| The model | 75  (68, 82) | 85  (79, 90) | 3.75  (2.86, 5.52) | 0.22  (0.14, 0.32) |
| 2D-SWE | 61  (50, 71) | 71  (61, 79) | 1.99  (1.60, 2.40) | 0.53  (0.40, 0.80) |
| **CHC** |  |  |  |  |
| The model | 69  (57, 80) | 83  (70, 93) | 2.43  (1.70, 3.83) | 0.23  (0.08, 0.40) |
| 2D-SWE | 72  (55, 85) | 81  (64, 93) | 2.77  (2.10, 3.60) | 0.25  (0.10, 0.60) |
| **MASLD** |  |  |  |  |
| The model | 60  (44, 74) | 95  (91, 98) | 6.91  (4.46, 11.89) | 0.25  (0.09, 0.42) |
| 2D-SWE | 54  (37, 71) | 97  (91, 99) | 5.42  (4.50, 6.50) | 0.16  (0.05, 0.50) |

Note.­Data in parentheses are 95% CIs. 2D-SWE, two-dimensional shear wave elastography; CHB, chronic hepatitis B; CHC, chronic hepatitis C; MASLD, metabolic dysfunction associated steatotic liver disease; PPV, positive predictive value; NPV, negative predictive value; LR, likelihood ratio.

Supplementary Table 7. Diagnostic performances of a deep learning-based radiomics model (DLRE-X ) and 2D-SWE in SSI, GE and Mindray sub-groups under the external test set.

|  | **PPV**  **%** | **NPV**  **%** | **LR+** | **LR-** |
| --- | --- | --- | --- | --- |
| SSI |  |  |  |  |
| The model | 50  (38, 62) | 90  (85, 95) | 3.62  (2.55, 5.50) | 0.39  (0.23, 0.56) |
| 2D-SWE | 49  (35, 63) | 93  (86, 97) | 3.49  (2.80, 4.30) | 0.28  (0.10, 0.60) |
| GE |  |  |  |  |
| The model | 79  (73, 85) | 85  (78, 91) | 3.66  (2.79, 5.19) | 0.17  (0.10, 0.25) |
| 2D-SWE | 78  (68, 86) | 72  (62, 80) | 3.51  (3.00, 4.10) | 0.39  (0.20, 0.60) |
| Mindray |  |  |  |  |
| The model | 71  (50, 90) | 95  (89, 100) | 8.12  (4.16, 29.25) | 0.19  (0.00, 0.40) |
| 2D-SWE | 37  (19, 58) | 92  (73, 99) | 1.91  (1.30, 2.80) | 0.30  (0.08, 1.10) |

Note.­Data in parentheses are 95% CIs. 2D-SWE, two-dimensional shear wave elastography; SSI, SuperSonic Imagine; GE, General Electric; PPV, positive predictive value; NPV, negative predictive value; LR, likelihood ratio.

Supplementary Table 8. Diagnostic performances of single- and dual-input of the deep learning-based radiomics model (DLRE-X ) in the region subgroup

|  | **N** | **Prevalence** | **AUC** | **Sensitivity**  **%** | **Specificity**  **%** | **PPV**  **%** | **NPV**  **%** | **LR+** | **LR-** |
| --- | --- | --- | --- | --- | --- | --- | --- | --- | --- |
| **China** | | | |  |  |  |  |  |  |
| The model | 221 | 40.2  (33.8, 46.8) | 0.87  (0.83, 0.91) | 82  (75, 88) | 79  (73, 84) | 72  (65, 79) | 87  (82, 92) | 3.87  (4.29, 3.2) | 0.23  (0.18, 0.24) |
| ConvNeXt | 221 | 40.2  (33.8, 46.8) | 0.78*  (0.73, 0.84) | 60  (51, 68) | 86  (80, 91) | 74  (64, 83) | 76  (70, 82) | 4.14  (4.84, 3.34) | 0.47  (0.38, 0.52) |
| **Japan** | | | |  |  |  |  |  |  |
| The model | 99 | 36.4  (26.7, 46.0) | 0.91  (0.85, 0.95) | 83  (72, 93) | 83  (74, 90) | 73  (60, 85) | 90  (83, 96) | 4.77  (6.58, 6.22) | 0.2  (0.15, 0.17) |
| ConvNeXt | 99 | 36.4  (26.7, 46.0) | 0.93  (0.89, 0.97) | 78  (67, 89) | 90  (83, 96) | 82  (70, 93) | 88  (81, 94) | 8.17  (8.41, 14.17) | 0.25  (0.28, 0.18) |
| **Europe** | | | |  |  |  |  |  |  |
| The model | 75 | 24.0  (14.1, 33.9) | 0.91  (0.84, 0.97) | 83  (67, 96) | 82  (74, 91) | 60  (43, 76) | 94  (88, 98) | 4.75  (3.68, 4.36) | 0.2  (0.27, 0.24) |
| ConvNeXt | 75 | 24.0  (14.1, 33.9) | 0.92  (0.85, 0.97) | 78  (61, 93) | 89  (82, 96) | 70  (52, 88) | 93  (86, 98) | 7.39  (7.31, 6.85) | 0.25  (0.11, 0.21) |

Note.­Data in parentheses are 95% CIs. AUC of dual-input was statistically compared with AUC of single-input in each country/region using the Delong test (*P < 0.05, **P < 0.01, ***P < 0.001). N, number of patients; AUC, area under the receiver characteristics curve; PPV, positive predictive value; NPV, negative predictive value; LR, likelihood ratio.

Supplementary Table 9. Diagnostic performances of single- and dual-input of the deep learning-based radiomics model (DLRE-X ) in the etiolgoy subgroup

|  | **N** | **Prevalence** | **AUC** | **Sensitivity**  **%** | **Specificity**  **%** | **PPV**  **%** | **NPV**  **%** | **LR+** | **LR-** |
| --- | --- | --- | --- | --- | --- | --- | --- | --- | --- |
| **CHB** | | | |  |  |  |  |  |  |
| The model | 195 | 44.1  (37.1, 51.1) | 0.87  (0.82, 0.91) | 83  (76, 89) | 78  (72, 85) | 75  (67, 82) | 85  (79, 90) | 3.75  (3.49, 4.73) | 0.22  (0.28, 0.25) |
| ConvNeXt | 195 | 44.1  (37.1, 51.1) | 0.77*  (0.72, 0.83) | 59  (51, 68) | 84  (79, 90) | 75  (66, 83) | 72  (66, 79) | 3.8  (6.17, 5.07) | 0.48  (0.37, 0.49) |
| **CHC** | | | |  |  |  |  |  |  |
| The model | 71 | 47.9  (36.0, 59.8) | 0.86  (0.78, 0.93) | 85  (75, 94) | 65  (51, 77) | 69  (57, 80) | 83  (70, 93) | 2.43  (3.43, 2.57) | 0.23  (0.19, 0.21) |
| ConvNeXt | 71 | 47.9  (36.0, 59.8) | 0.86  (0.77, 0.92) | 76  (63, 88) | 73  (60, 85) | 72  (59, 84) | 77  (65, 88) | 2.83  (4.26, 3.73) | 0.32  (0.21, 0.22) |
| **MASLD** | | | |  |  |  |  |  |  |
| The model | 129 | 17.8  (11.1, 24.5) | 0.9  (0.82, 0.96) | 78  (64, 92) | 89  (83, 93) | 60  (44, 74) | 95  (91, 98) | 6.91  (3.7, 6.17) | 0.25  (0.41, 0.17) |
| ConvNeXt | 129 | 17.8  (11.1, 24.5) | 0.94  (0.88, 0.98) | 78  (63, 92) | 96  (93, 99) | 82  (67, 94) | 95  (92, 98) | 20.74  (16.51, 20.57) | 0.23  (0.04, 0.25) |

Note.­Data in parentheses are 95% CIs. AUC of dual-input was statistically compared with AUC of single-input in each etiolgoy using the Delong test (*P < 0.05, **P < 0.01, ***P < 0.001). N, number of patients; AUC, area under the receiver characteristics curve; PPV, positive predictive value; NPV, negative predictive value; LR, likelihood ratio; CHB, chronic hepatitis B; CHC, chronic C, MASLD, non-alcoholic fatty liver disease.

Supplementary Table 10. Diagnostic performances of single- and dual-input of the deep learning-based radiomics model (DLRE-X ) in the device manufacturer subgroup

|  | **N** | **Prevalence** | **AUC** | **Sensitivity**  **%** | **Specificity**  **%** | **PPV**  **%** | **NPV**  **%** | **LR+** | **LR-** |
| --- | --- | --- | --- | --- | --- | --- | --- | --- | --- |
| **SSI** | | | |  |  |  |  |  |  |
| The model | 148 | 21.6  (14.9-28.3) | 0.84  (0.78, 0.9) | 69  (54, 81) | 81  (75, 87) | 50  (38, 62) | 90  (85, 95) | 3.62  (4.34, 3.48) | 0.39  (0.31, 0.26) |
| ConvNeXt | 148 | 21.6  (14.9-28.3) | 0.74  (0.64, 0.84) | 56  (40, 71) | 90  (85, 94) | 60  (44, 74) | 88  (83, 93) | 5.44  (9.44, 5.22) | 0.49  (0.38, 0.45) |
| **GE** | | | |  |  |  |  |  |  |
| The model | 196 | 50.5  (43.4, 47.6) | 0.9  (0.86, 0.93) | 87  (81, 92) | 76  (69, 83) | 79  (73, 85) | 85  (78, 91) | 3.66  (4.16, 4.32) | 0.17  (0.13, 0.11) |
| ConvNeXt | 196 | 50.5  (43.4, 47.6) | 0.87  (0.83, 0.91) | 68  (60, 75) | 90  (84, 94) | 87  (80, 93) | 73  (66, 79) | 6.56  (6.0, 8.42) | 0.36  (0.29, 0.3) |
| **Mindray** | | | |  |  |  |  |  |  |
| The model | 51 | 23.5  (11.5, 35.6) | 0.88  (0.76, 0.99) | 83  (64, 100) | 90  (81, 97) | 71  (50, 90) | 95  (88, 100) | 8.12  (7.36, 7.5) | 0.19  (0.22, 0.0) |
| ConvNeXt | 51 | 23.5  (11.5, 35.6) | 0.87  (0.76, 0.98) | 83  (64, 100) | 77  (66, 88) | 53  (33, 72) | 94  (87, 100) | 3.61  (4.69, 4.5) | 0.22  (0.24, 0.0) |

Note.­Data in parentheses are 95% CIs. AUC of dual-input was statistically compared with AUC of single-input in each device manufacturer using the Delong test (*P < 0.05, **P < 0.01, ***P < 0.001). N, number of patients; AUC, area under the receiver characteristics curve; PPV, positive predictive value; NPV, negative predictive value; LR, likelihood ratio; SSI, SuperSonic Imagine; GE, General Electric.

Supplementary Table 11. Optimal LSM cut-offs of 2D-SWE in different sub-groups under the external test set.

| Type | Subgroups | Optimal LSM cut-offs (kPa) |
| --- | --- | --- |
| Region | China | 8.60 |
|  | Japan | 9.56 |
|  | Europe | 8.80 |
| Etiologies | CHB | 8.60 |
|  | CHC | 8.36 |
|  | MASLD | 9.02 |
| Manufacturer | SSI | 8.68 |
|  | GE | 7.09 |
|  | Mindray | 10.66 |

Note.­Optimal LSM cut-offs were calculated by the Youden index in each subgroup. LSM, liver stiffness measurement; CHB, chronic hepatitis B; CHC, chronic hepatitis C; MASLD, metabolic dysfunction associated steatotic liver disease. SSI, SuperSonic Imagine; GE, General Electric.
