# Supplementary material for: Deep learning radiomics of elastography for diagnosing compensated advanced chronic liver disease: an international multicenter study
Source: Vis Comput Ind Biomed Art. 2025 Aug 15;8:19. doi: 10.1186/s42492-025-00199-6 (PMC12354435; doi:10.1186/s42492-025-00199-6)
Supplement: Supplementary file 2 — Supplementary Material 2. [file 42492_2025_199_MOESM2_ESM.docx]

**Supplementary materials**

**Supplementary materials and methods**

2D-SWE measurements.

*Machines*

2D-SWE from three manufacturers were used in the study, including SuperSonic Imagine, SSI, France, Logic E9, GE, American and Resona7, Mindray, China. Three 2D-SWE manufacturers (SSI, GE and Mindray) were included in our study, and only one of them was used to scan each patient.

*2D-SWE procedures*

Three convex array probes from three manufacturers were taken in our study, including SC6-1 from 2D-SWE.SSI, C1-6 from 2D-SWE.GE and SC6-1U from 2D-SWE.Mindray. Given the differences of the 2D-SWE technique details in manufacturers, the 2D-SWE sampling frame and Q-Box settings were different. For SSI and Mindray, the 2D-SWE sampling frame size is 4 cm×3 cm, and the diameter of the Q-Box is set to 2 cm (Suppl. Fig. 1A&C). But for GE, the sampling frame is 2 cm×10 cm and a Q-Box diameter of 1 cm (Suppl. Fig. 1B). According to the latest WFUMB guidelines, 3-5 measurements should be obtained for 2D-SWE measurements. The 2D-SWE ROI was positioned 15-20 mm below the liver capsule, avoiding large blood vessels, bile ducts and masses. We recommended five measurements for each patient and at least three measurements were obtained. The median value of multiple measurements is obtained as the liver stiffness value.

*Quality control*

Operators from every center performed 2D-SWE examinations. All operators have more than three years 2D-SWE examinations experience and were blinded to pathological results. One experienced operator(XL) with more than five years 2D-SWE examination experience reviewed all images independently. Measurements were considered a failure or unqualified when no more than two-thirds of the signal filled in the 2D SWE region of interest or when the large vessels and biliary tracts were not avoided for every acquisition. Unqualified images were excluded. If one patient had less than three qualified images, the whole case was excluded.

*2D-SWE cut-offs*

The 2D-SWE cut-offs of the training set were calculated by the Youden index. Then, it was tested in the internal and external test sets as well as regions, etiologies and manufacturers sub-sets to calculate the accuracy, sensitivity, specificity, positive and negative predictive values, and positive and negative likelihood ratios. Furthermore, we calculated each group and sub-group optimal cut-offs (named specific cut-offs).

Clinical data collection

The demographic and clinical data of eligible patients (sex, age, body mass index, serological examinations, etc.) were collected for analysis. Serological examinations were performed within two weeks of 2D-SWE. The alanine aminotransferase (ALT), albumin (ALB), total bilirubin (TB), direct bilirubin (DB), indirect bilirubin (IB), gamma-glutamyl transpeptidase (GGT), and prothrombin activity (PT) were recorded.

Pathological Evaluation

Patients participating in our study underwent liver biopsy after 2D-SWE measurements. A 16- or 18-G needle (Bard Magnum, GA, USA) was used under the guidance of ultrasound. Pathological results were interpreted by experienced pathologists who have more than three years experience in Hepatic pathology in participated centers. The fibrosis degrees were evaluated by METAVIR (patients for CHB or CHC) or Ishak (patients for MASLD). cACLD was defined as ≥F3 (METAVIR scoring systems) or ≥S4 (Ishak scoring systems).

Establishing DLRE-X .

*The input layer selection of DLRE-X*

A semi-automatic image cropping algorithm was used for this procedure. The center point of the pseudo-color display area on each 2D-SWE image was manually selected. Then, the ROI for DLRE-X input was automatically generated. This semi-automatic procedure was performed on all 2D-SWE images. Therefore, there were 3-5 ROIs obtained as DLRE-X inputs for each patient, which minimized the inter-operator variability for input ROI definition. The code of the cropping algorithm is available at https://github.com/samadhi-fire/DLRE-3.0.

ConvNeXt is a neural network architecture based purely on convolutional operations. By incorporating strategies such as large-kernel depthwise convolution and layer normalization, it achieves performance comparable to Transformers. The model adopts a hierarchical design, consisting of three main parts: the input layer (Stem), four progressive downsampling stages (Stage 1~4), and a classification head. The input is an RGB image (3×224×224), and the output is a binary probability vector. The detailed structure and data transformation process are as follows:

**1. Input Preprocessing and Stem Layer**

The input image first undergoes normalization (pixel values scaled to zero mean and unit variance) before entering the stem layer. This layer consists of a single 4×4 convolutional kernel with a stride of 4 and an output channel count of 128, followed by layer normalization (LayerNorm). This process compresses the input dimensions from 3×224×224 to 96×56×56, significantly reducing spatial resolution while preliminarily expanding the feature channels to establish a foundation for high-dimensional feature extraction in subsequent layers. Notably, ConvNeXt abandons the traditional CNN practice of using BatchNorm, instead adopting LayerNorm—consistent with Transformers—to enhance training stability.

**2. Hierarchical Feature Extraction Stages (Stage 1~4)**

The main body of the model comprises four stages, each containing multiple ConvNeXt blocks and a downsampling module. Through these stages, the channel count is progressively doubled (128→256→512→1024) while the resolution is reduced (56×56→28×28→14×14→7×7), enabling multi-scale feature encoding.

Each ConvNeXt block consists of: 1) A 7×7 depthwise separable convolution, simulating the wide receptive field characteristic of ViT's local window attention mechanism. 2) LayerNorm applied to the convolutional output. 3) An inverted bottleneck structure: First, a 1×1 convolution expands the channel count by 4 (e.g., 128→512), followed by GELU activation, then another 1×1 convolution compresses it back to the original channel count, forming a nonlinear transformation. 4) A residual connection that adds the transformed result to the input, mitigating gradient vanishing.

The progressive downsampling module at the end of each stage uses LayerNorm followed by a 2×2 convolution with stride 2 to halve the resolution while doubling the channel count. For example, Stage 1 takes an input of 128×56×56, processes it through 3 ConvNeXt blocks, and outputs 256×28×28 after downsampling. Stage 3 employs 9 blocks for deeper feature extraction, while other stages use 3 blocks to balance computational efficiency and model capacity.

3. LSM (Liver Stiffness Measurement) Fusion and Prediction Output

After the final stage outputs a 1024×7×7 feature map, the model applies global average pooling (GAP) to compress it into a 1024×1×1 vector, representing the extracted useful information from the image. This reduced feature vector is then concatenated with the LSM value, forming a joint feature vector that allows the model to simultaneously analyze information from both elastography and LSM measurements.

A multilayer perceptron (MLP) processes this joint feature vector, automatically learning and integrating information from different modalities. The final output layer generates a probability distribution (of dimension 2) corresponding to the target classes, providing a quantitative predictive assessment for cACLD (compensated advanced chronic liver disease).

**Supplementary results.**

Baseline characteristics

Between January 2012 and December 2019, a total of 2591 potentially eligible CLD patients were enrolled from 17 centers in East Asia and Europe. Among them, 654 patients were excluded based on our exclusion criteria, resulting in 1937 patients with 9472 2D-SWE images selected for analysis in this study (Figure 2a). According to our study design, 1233 and 309 patients from China and Japan were randomly allocated into the training and internal test sets, respectively. The remaining 395 patients went to the external test set. Detailed distribution of enrolled patients regarding the regions, etiologies, manufacturers, and participated centers is shown in Figure 2b-d and Supplementary Table 1. The numbers of 2D-SWE images in training, internal test set and external test sets were 6150, 1543 and 1779, respectively. In region subgroups, there were 976, 493 and 310 2D-SWE images acquired from China, Japan and Europe, respectively. In etiology subgroups, there were 847, 348 and 584 2D-SWE images acquired from CHB, CHC and MASLD patients, respectively. In manufacturer subgroups, there were 673, 852 and 254 2D-SWE images acquired by SSI, GE and Mindray systems, respectively.

Overall benefit comparisons between DLRE-X and 2D-SWE

DCA curves of DLRE-X and 2D-SWE presented in supplementary figure 3 are for predicting the probability of cACLD in training set a)，internal test set b), and external test set c). The horizontal and vertical axes represent the threshold probability and net benefit, respectively. The lines between the horizontal axis and vertical axis display the benefit of different diagnose methods. The DCA curves show that if the threshold probability is between 10% and 90% with purple area, using DLRE-X and 2D-SWE in the current study to predict cACLD risk could add more benefit when compared with either the treat-all or the treat-none strategies. DLRE-X can provide patients more benefits if the threshold probability is in the range of 30% to 80%, compared with 2D-SWE, with the red area in supplementary figure 3.

Single- versus dual-input performance of DLRE-X

The results of single- and dual-input evaluations are shown in Supplementary Table 4. In the training and internal test sets, single- and dual-input of the model achieved similar AUCs. However, in the external test set, dual-input improved AUC compared with single-input (0.89; 95%CI: 0.86, 0.91; vs. 0.83; 95%CI: 0.79, 0.86; *P*=.02).

Single- versus dual-input performance of DLRE-X in subgroups

Single versus dual-input performance of DLRE-X in three regions/etiologies/manufactures subgroups were shown in Supplementary Table 8-10. Overall speaking, dual-input DLRE-X achieved higher or comparable diagnostic accuracy, especially in China and chronic hepatitis B subgroup, which dual-input provided statistically higher AUCs than single input.

Failure analysis for the DLRE-X model

All false-negative and false-positive cases given by DLRE-X , including both 2D-SWE images and corresponding LSM values were reviewed. However, none of us could find any obvious pattern other than that those wrong cases were really close to the correct cases. A few examples were listed in supplementary figure 4.

**Supplementary discussion.**

More interestingly, many of the false positive and false negative cases given by single and dual-input methods did not coincide. In our opinion, DLRE-X was designed to improve the accuracy and robustness of 2D-SWE examinations for cACLD diagnosis by fully quantifying and analyzing the available data. However, 2D-SWE examination itself has physical limitations. Even if all of its diagnostic potential is fully utilized, it cannot be 100% correct for the large sample size from different hospitals in multiple countries/regions, when using devices from different manufacturers.

**References**

44 He, Kaiming, et al. "Deep residual learning for image recognition." Proceedings of the IEEE conference on computer vision and pattern recognition. 2016.

45 Liu, Ze, et al. "Swin transformer: Hierarchical vision transformer using shifted windows." arXiv preprint arXiv:2103.14030 (2021).

46 Xie, Saining, et al. "Aggregated residual transformations for deep neural networks." Proceedings of the IEEE conference on computer vision and pattern recognition. 2017.

47 Sandler, Mark, et al. "Mobilenetv2: Inverted residuals and linear bottlenecks." Proceedings of the IEEE conference on computer vision and pattern recognition. 2018.

48 Ba, Jimmy Lei, Jamie Ryan Kiros, and Geoffrey E. Hinton. "Layer normalization." arXiv preprint arXiv:1607.06450 (2016).

49 Hendrycks, Dan, and Kevin Gimpel. "Gaussian error linear units (gelus)." arXiv preprint arXiv:1606.08415 (2016).
