# Supplementary material for: Deep learning radiomics of elastography for diagnosing compensated advanced chronic liver disease: an international multicenter study
Source: Vis Comput Ind Biomed Art. 2025 Aug 15;8:19. doi: 10.1186/s42492-025-00199-6 (PMC12354435; doi:10.1186/s42492-025-00199-6)
Supplement: Supplementary file 3 — Supplementary Material 3. [file 42492_2025_199_MOESM3_ESM.docx]

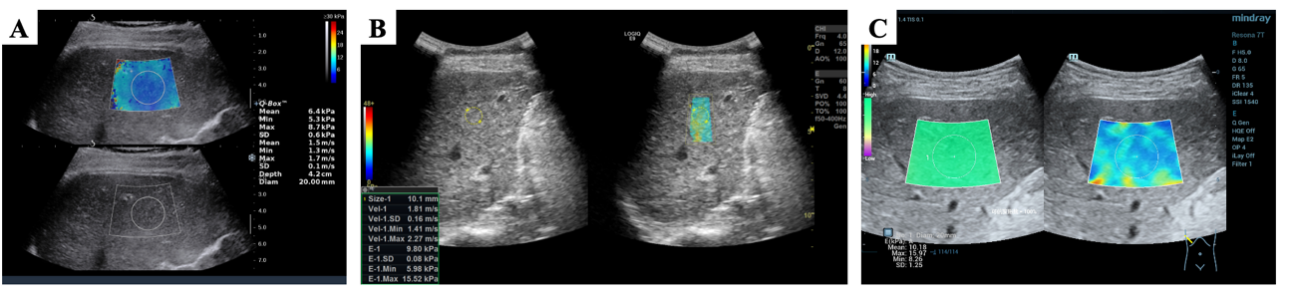


**Supplementary Figure 1. Examples of 2D-SWE images from three manufacturers.** (A) SSI; (B) GE; (C) Mindray. 2D-SWE = two-dimensional shear wave elastography, SSI = SuperSonic Imagine, GE = General Electric


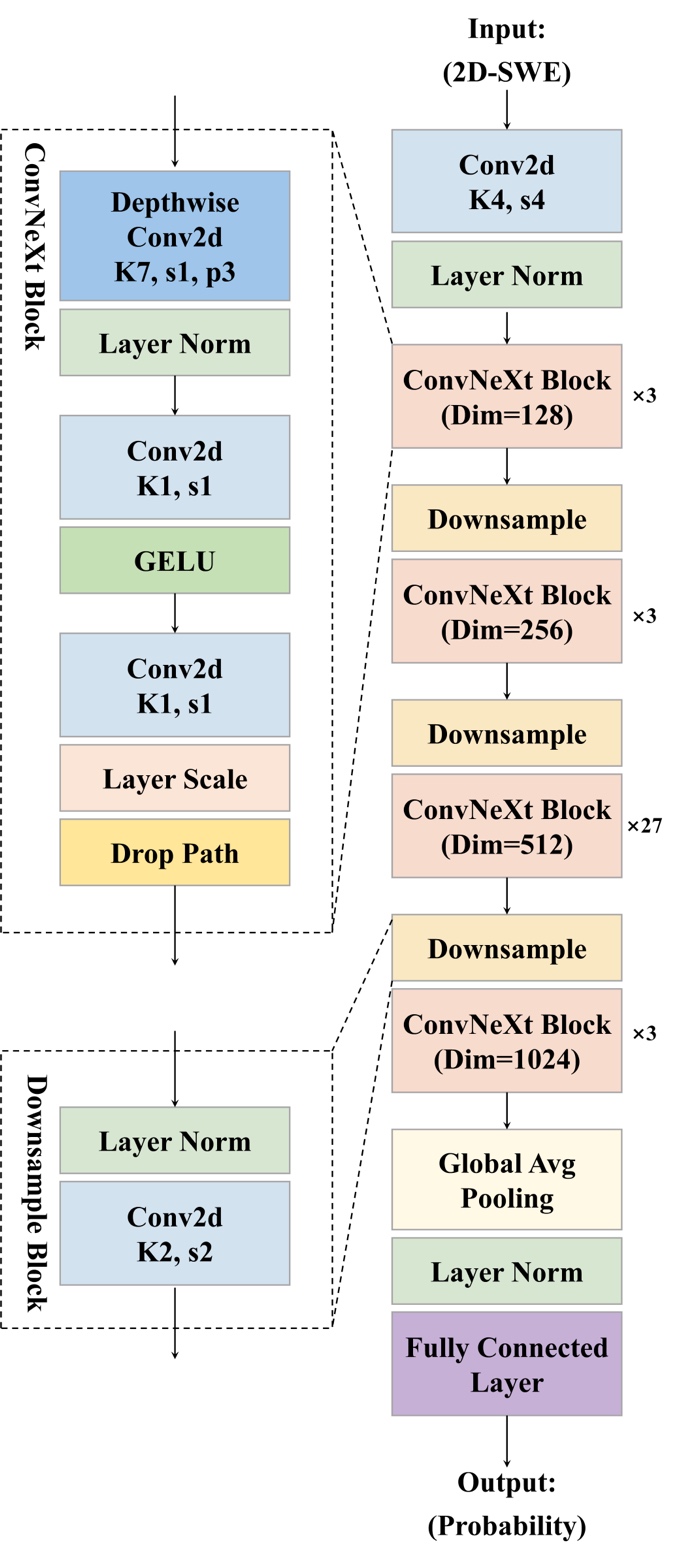


**Supplementary Figure 2. Illustration of the detailed architecture of the ConvNeXt network, showing the design of ConvNeXt block and the downsample block.** Conv2d = 2D convolutional Layer, Dim = dimension, Global Avg Pooling = global average pooling.


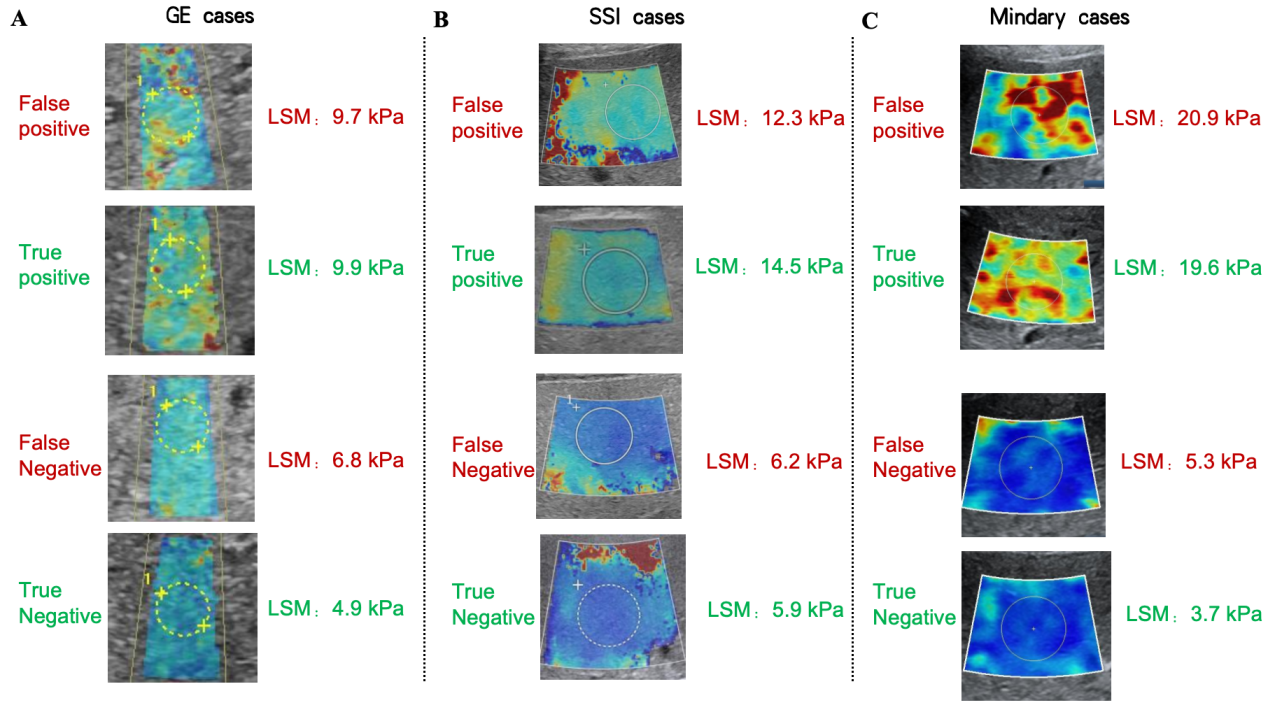


**Supplementary Figure 3. Representative failure cases in the external test cohort given by the model.** (A) Cases of GE; (B) Cases of SSI; (C) Cases of Mindray. GE = General Electric; SSI = SuperSonic Imagine.


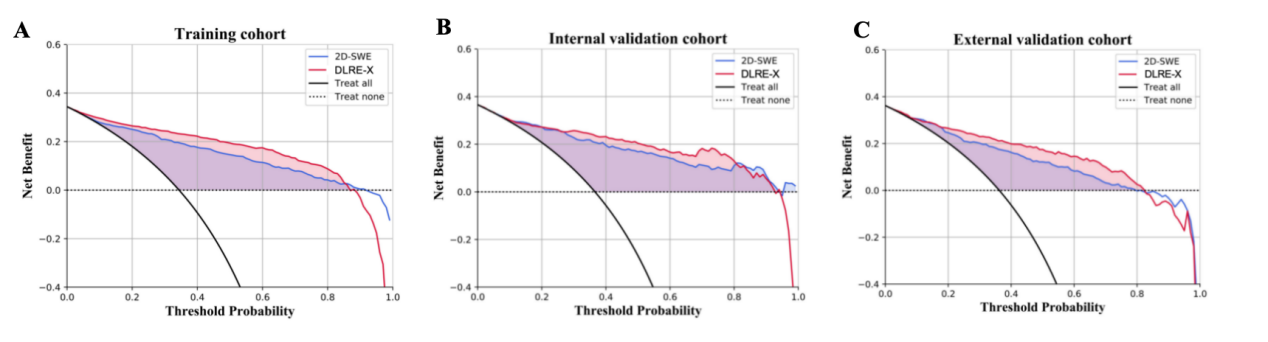
**Supplementary Figure 4. Comparison of DCA curves between the deep learning-based radiomics model DLRE-X and 2D-SWE.** (a) DCA curves of DLRE-X and 2D-SWE in the training sets; (b) DCA curves of DLRE-X and 2D-SWE in the internal test sets; (c) DCA curves of DLRE-X and 2D-SWE in the external test sets. DCA = decision curves area, DLRE-X = version 3.0 deep learning radiomics of elastography, 2D-SWE = two-dimensional shear wave elastography.
